# Isolation and identification of compounds from the resinous exudate of *Escallonia illinita* Presl. and their anti-oomycete activity

**DOI:** 10.1186/s13065-019-0516-8

**Published:** 2019-01-28

**Authors:** Iván Montenegro, Elizabeth Sánchez, Enrique Werner, Patricio Godoy, Yusser Olguín, Nelson Caro, Nicole Ehrenfeld, Alejandro Madrid

**Affiliations:** 1 Escuela de Obstetricia y Puericultura, Facultad de Medicina, Campus de la Salud, Universidad de Valparaíso, Angamos 655, Reñaca, 2520000 Viña del Mar, Chile; 2 Centro de Biotecnología, Dr. Daniel AlKalay Lowitt, Universidad Técnica Federico Santa María, Avda. España 1680, 2340000 Valparaiso, Chile; 3 Departamento de Ciencias Básicas, Campus Fernando May Universidad del Biobío, Avda. Andrés Bello s/n casilla 447, 3780000 Chillán, Chile; 4 Instituto de Microbiología Clínica, Facultad de Medicina, Universidad Austral de Chile, Los Laureles s/n, Isla Teja, 5090000 Valdivia, Chile; 5 Instituto de Investigación Interdisciplinar en Ciencias Biomedicas SEK (I3CBSEK), Facultad de Ciencias de la Salud, Universidad SEK, Fernando Manterola 0789, 7500000 Santiago, Chile; 6 Centro de Investigación Australbiotech, Universidad Santo Tomás, Avda. Ejército 146, 8320000 Santiago, Chile; 7 Departamento de Química, Facultad de Ciencias Naturales y Exactas, Universidad de Playa Ancha, Avda. Leopoldo Carvallo 270, Playa Ancha, 2340000 Valparaiso, Chile

**Keywords:** *Escallonia illinita*, Resinous exudates, Anti-oomycete activity, *Saprolegnia* sp.

## Abstract

The resinous exudates from *Escallonia illinita* by products was characterized by FT-IR, NMR and HRMS. Six compounds were isolated and identified as follows: 1,5-diphenylpent-1-en-3-one (**1**), 4-(5-hydroxy-3,7-dimethoxy-4-oxo-4*H*-chromen-2-yl)phenyl acetate (**2**), pinocembrin (**3**), kaempferol 3-*O*-methylether (**4**), (3*S*,5*S*)-(*E*)-1,7-diphenylhept-1-ene-3,5-diol (**5**) and the new diarylheptanoid (3*S*,5*S*)-(*E*)-5-hydroxy-1,7-diphenylhept-1-en-3-yl acetate (**6**). The anti-oomycete potential of the resinous exudate, as well as the main compounds, was tested in vitro against *Saprolegnia parasitica* and *Saprolegnia australis*. The resinous exudate showed a strong anti-oomycete activity. In addition, the compounds **6**, **1** and **3** demonstrated significant inhibition of *Saprolegnia* strains development. These findings strongly suggest that *E. illinita* is a potential biomass that could be used as a natural anti-oomycete product.

**Electronic supplementary material:**

The online version of this article (10.1186/s13065-019-0516-8) contains supplementary material, which is available to authorized users.

## Introduction

The genus *Saprolegnia* belongs to the group of heterotrophs known as oomycetes, commonly called water molds, which are saprophytes or parasites targeting a wide range of hosts [1]. They are a very important fish pathogen, especially on catfish, salmon and trout species, and that attacks even crustaceans and amphibians of hatchery [2–4]. As a consequence diseases caused by these oomycetes produce considerable losses in world aquaculture [5, 6], especially on salmon farming because it infects adults and eggs [7]. *Saprolegnia* sp. has traditionally been controlled by commercial fungicides (malachite green, formalin, hydrogen peroxide and bronopol) [8, 9]. However, the use of these fungicides has caused serious problems such as the appearance of highly resistant strains, and the contamination of environment [10, 11]. The intrinsic need to seek and develop new oomycides is not only due to these fungicide-resistant strains, but also due to the demand for organically grown foods, which is rapidly increasing because of concerns about human health and environmental quality [12]. Thus, there is a growing trend towards using natural products, regarded as environmentally friendly alternatives to synthetic fungicides or oomycides for the protection of the fish farming against water molds caused by members of the genus *Saprolegnia.* Little information is available in the literature on anti-oomycete activity of natural products against *Saprolegnia* sp. Some flavonoids [13], chalcones [14–16], phenylpropanoids [17], essential oil [18, 19] and seaweed extracts [20] have effect against these oomycetes.

The resinous shrub *Escallonia illinita* Presl., which is widely distributed in south central of Chile, is widely used by traditional Chilean medicine “barraco”. It was used as folk medicine for immune-modulation, anti-tumor, anti-fungal and anti-bacterial [21]. Previous studies on this plant revealed that the aqueous and hydroalcoholic extracts of *E. illinita* showed significant anti-viral, anti-fungal, anti-bacterial and anti-parasitic activities in vitro [22, 23]. To further investigate the constituents and screen the bioactive constituents from the resinous exudate of this herbal medicine, a phytochemical study was performed that resulted in the isolation of one new compound, along with five known components. Herein, we report the isolation, structural elucidation, and anti-oomycete activity of compounds **1**–**6**.

## Experimental section

Unless otherwise stated, all chemical reagents purchased (Merck, Darmstadt, Germany or Aldrich, St. Louis, MO, USA) were of the highest commercially available purity and were used without previous purification. IR spectra were recorded as thin films in a FT-IR Nicolet 6700 spectrometer (Thermo Scientific, San Jose, CA, USA) and frequencies are reported in cm^−1^. ^1^H and ^13^C spectra were recorded on a Bruker Avance 400 Digital NMR spectrometer (Bruker, Rheinstetten, Germany), operating at 400.1 MHz for ^1^H and 100.6 MHz for ^13^C. Chemical shifts are reported in *δ* ppm and coupling constants (*J*) are given in Hz. HREIMS were measured on Thermo Finnigan MAT95XL mass spectrometers. Silica gel (Merck 200–300 mesh) was used for C.C. and silica gel plates HF 254 for TLC. TLC spots were detected by heating after spraying with 25% H_2_SO_4_ in H_2_O.

## Plant material

Aerial parts of *E. illinita* were collected in Limache, Valparaíso Region, Chile, in November of 2017. A voucher specimen (VALPL 2155) was deposited at the VALP Herbarium, Department of Biology, Universidad de Playa Ancha, Valparaíso, Chile.

## Extraction and isolation

Fresh *E. illinita* (800 g) aerial parts were extracted with cold dichloromethane (5 L) at room temperature for 45 s that produced (12.3 g) of the resinous exudate with w/w yield of 15.38%. Later, the resinous exudate (5.00 g) was fractionated by column chromatography on silica gel using *n*-hexane–ethyl acetate (100:0 to 0:100, v/v) to obtain five major Fractions A, B, C, D and E, respectively. Fr. A (1.26 g) was further purified by column chromatography on silica gel eluting with *n*-hexane–ethyl acetate (8:2, v/v) to give compounds **1** (71.50 mg) and **2** (64.59 mg). Fr. B (1.08 g) was separated by column chromatography on silica gel eluting with *n*-hexane–ethyl acetate (7:3, v/v) to three fractions were obtained: fraction I (120.59 mg) of compound **3**, fraction II (419.91 mg), a mixture of compounds, subsequently derivatized and fraction III (188.61 mg) of compound **4**. Fr. C (912.03 mg) was subjected to column chromatography on silica gel eluting with *n*-hexane–ethyl acetate (9:1, v/v) to give compounds **3** (193.75 mg) and **4** (40.36 mg). Compound **5** (152.60 mg) was precipitated from Fr. D (436 mg) using MeOH. Fr. E (717 mg) was purified by column chromatography on silica gel eluting with *n*-hexane–ethyl acetate (4:6, v/v) to give compound **6** (127.40 mg).

## Structural elucidation of natural compounds **1**–**6**

### (*E*)-1,5-Diphenylpent-1-en-3-one (**1**)

White solid. m.p.: 54–55 °C. IR ν/cm^−1^: 2928 (C–H), 1625 (C=O), 1605 (C=C). ^1^H NMR (400 MHz, CDCl_3_) *δ*/ppm: 7.46 (d, *J *= 7.0 Hz, 2H, H-2′ and H-6′); 7.34 (m, 3H, H-3′, H-4′ and H-5′); 7.21 (m, 4H, H-2″, H-3″, H-5″ and H-6″); 6.90 (m, 2H, H-1 and H-4″); 6.28 (b.d., *J *= 15.4 Hz, 1H, H-2); 2.95 (m, 4H, H-4 and H-5). ^13^C NMR (100 MHz, CDCl_3_) *δ*/ppm: 199.4 (C-3); 141.4 (C-1); 141.2 (C-1″); 136.0 (C-1′); 129.5 (C-3′ and C5′); 129.2 (C-4′); 128.5 (C-3″ and C-5″); 1128.4 (C-2″ and C-6″); 127.2 (C-2′ and C-6′); 126.6 (C-4″); 126.1 (C-2); 42.3 (C-4); 30.2 (C-5). HREIMS: M+H ion m/z 237.3083 (calcd. for C_17_H_16_O: 236.3145).

### 4-(5-Hydroxy-3,7-dimethoxy-4-oxo-4*H*-chromen-2-yl)phenyl acetate (**2**)

Colorless solid. m.p.: 165–166 °C. IR ν/cm^−1^: 3280 (O–H), 1670 (C=O), 1610 (C=C), 1310 (O–C). 1H NMR (400 MHz, CDCl_3_) *δ*/ppm: 12.55 (s, 1H, OH), 8.12 (s, 2H, H-2′ and H-6′), 7.26 (s, 2H, H-3′ and H-5′), 6.45 (s, 1H, H-8), 6.37 (s, 1H, H-6), 3.88 (s, 6H, 2xOCH_3_), 2.35 (s, 3H, OAc). ^13^C NMR (100 MHz, CDCl_3_) *δ*/ppm: 178.9 (C-4), 169.0 (OAc), 165.6 (C-5), 156.8 (C-10), 154.9 (C-2), 152.4 (C-4′), 139.7 (C-3), 129.8 (C-2′ and C-6′), 128.0 (C-1′), 121.9 (C-3′ and C-5′), 106.2 (C-9), 98.0 (C-6), 92.2 (C-8), 60.4 (OCH_3_); 55.8 (OCH_3_), 21.2 (CH_3_). HREIMS: M+H ion m/z 357.3325 (calcd. for C_19_H_16_O_7_: 356.3261).

### Pinocembrin (**3**)

Colorless solid. [α]D_20_ = − 45.3° (c = 0.9, acetone). m.p.: 190–191 °C. IR ν/cm^−1^: 3230 (O–H), 1660 (C=O), 1620 (C=C). ^1^H NMR (400 MHz, CDCl_3_) *δ*/ppm: 12.15 (s, 1H, OH), 9.83 (b.s., 1H, OH), 7.42 (m, 5H, H-2′, H-3′, H-4′, H-5′ and H-6′), 6.00 (s, 2H, H-6 and H-8), 5.40 (dd, *J *= 13.2 and *J *= 2.4 Hz, 1H, H-2), 3.10 (dd, *J *= 17.1 and *J *= 13.6 Hz, 1H, H-3α), 2.80 (dd, *J *= 17.1 and *J *= 2.6 Hz, 1H, H-3β). ^13^C NMR (100 MHz, CDCl_3_) *δ*/ppm: 196.8 (C-4), 167.3 (C-7), 165.3 (C-5), 164.9 (C-9), 140.0 (C-1′), 129.4 (C-3′, C-4′ and C-5′), 127.3 (C-2′ and C-6′), 103.1 (C-5), 96.8 (C-6), 95.9 (C-8), 79.9 (C-2); 43.6 (C-3). HREIMS: M+H ion m/z 257.2584 (calcd. for C_15_H_12_O_4_: 256.2534).

### Kaempferol 3-*O*-methylether (**4**)

White solid. m.p.: 271–272 °C. IR ν/cm^−1^: 3230 (O–H), 1660 (C=O), 1620 (C=C). ^1^H NMR (400 MHz, CDCl_3_) *δ*/ppm: 12.78 (s, 1H, OH), 8.02 (s, 2H, H-2′ and H-6′), 7.00 (s, 2H, H-3′ and H-5′), 6.49 (s, 1H, H-8), 6.25 (s, 1H, H-6), 3.85 (s, 3H, OCH_3_). ^13^C NMR (100 MHz, CDCl_3_) *δ*/ppm: 176.0 (C-4), 164.1 (C-7), 162.6 (C-4′), 160.6 (C-5), 158.0 (C-10), 149.5 (C-2), 136.8 (C-3), 131.1 (C-2′ and 6′), 122.5 (C-1′), 116.3 (C-3′ and C-5′), 103.0 (C-9), 98.3 (C-6), 94.5 (C-8), 60.2 (O–CH_3_). HREIMS: M+H ion m/z 301.3681 (calcd. for C_16_H_12_O_6_: 300.2629).

### (3*S*,5*S*)-(*E*)-1,7-Diphenylhept-1-ene-3,5-diol (**5**)

Colorless needles. m.p.: 75–77 °C. [α]_D_23 =+ 25.19° (c = 0.63, MeOH). IR ν/cm^−1^: 3540 (O–H), 1640 (C=C). ^1^H NMR (400 MHz, CDCl_3_) δ/ppm: 7.38 (d, J = 7.3 Hz, 2H, H-2′ and H-6′), 7.24 (m, 5H, H-3′, H-4′, H-5′, H-3″ and H-5″), 7.21 (m, 3H, H-2″, H-4″ and H-6″), 6.63 (d, *J *= 15.8 Hz, 1H, H-1), 6.27 (dd, *J *= 6.1 and *J *= 15.8 Hz 1H, H-2), 4.80 (b.s., 1H, OH), 4.67 (m, 1H, H-3), 4.03 (m, 1H, H-5), 2.81 (m, 1H, H-7α), 2.65 (m, 1H, H-7β), 2.49 (b.s., 1H, OH), 1.85 (m, 2H, H-4), 1.79 (m, 2H, H-6). ^13^C NMR (100 MHz, CDCl_3_) *δ*/ppm: 141.9 (C-1″), 135.6 (C-1′), 131.8 (C-2), 130.1 (C-1), 128.6 (C-3′ and C-5′), 128.5 (C-2″, C-3″, C-5″ and C-6″), 127.7 (C-4′), 126.5 (C-2′), 125.9 (C-4″), 70.7 (C-3), 68.9 (C-5), 42.6 (C-4); 39.2 (C-6), 32.1 (C-7). HREIMS: M+H ion m/z 283.3834 (calcd. for C_19_H_22_O_2_: 282.3768).

### (3*S*,5*S*)-(*E*)-5-Hydroxy-1,7-diphenylhept-1-en-3-yl acetate (**6**)

White needles. m.p: 89–91 °C. [α]_D_23 = + 25.09° (c = 0.63, MeOH). IR ν/cm^−1^: 3330 (O–H), 1690 (C=O), 1610 (C=C). ^1^H NMR (400 MHz, CDCl_3_) δ/ppm: 7.36 (d, *J *= 7.8 Hz, 2H, H-2′ and H-6′), 7.26 (m, 5H, H-3′, H-4′, H-5′, H-3″ and H-5″), 7.17 (m, 3H, H-2″, H-4″ and H-6″), 6.55 (d, *J *= 15.8 Hz, 1H, H-1), 6.07 (m, 1H, H-2), 5.68 (m, 1H, H-3), 3.53 (m, 1H, H-5), 2.97 (b.s., 1H, OH), 2.81 (m, 1H, H-7α), 2.65 (m, 1H, H-7β), 2.03 (s, 3H, CH_3_), 1.79 (m, 2H, H-4), 1.68 (m, 2H, H-6). ^13^C NMR (100 MHz, CDCl_3_) δ/ppm: 171.7 (OAc), 142.0 (C-1″), 135.9 (C-1′), 131.6 (C-1), 129.4 (C-3′ and C-5′), 128.6 (C-2′ and C-6′), 128.5 (C-2″, C-3″, C-5″ and C-6″), 128.3 (C-4′), 127.6 (C-2), 125.8 (C-4′’), 68.6 (C-3), 66.6 (C-5), 43.3 (C-4); 38.6 (C-6), 32.1 (C-7), 21.1 (COCH_3_). HREIMS: M+H ion m/z 325.4211 (calcd. for C_21_H_24_O_3_: 324.4134).

## Oomycete strain

Pure strains of *S. parasitica* and *S. australis* were received from the Cell Biology Laboratory, Faculty of medicine, Universidad de Valparaíso, placed on potato dextrose agar (PDA) slants, and stored at 4 °C. This pure strain was isolated from *Salmo salar* carp eggs [19].

## Minimum inhibitory concentration evaluation

The method used in this study for anti-oomycete activity assay was performed according to methods previously reported [19]. The resinous exudates and the compounds **1**–**6** were tested at 200.0, 150.0, 100.0, 50.0, 25.0, 12.5, 6.3, and 3.1 µg/L to find a preliminary minimum inhibitory concentration (MIC) interval. The MIC values were recorded visually on the basis of mycelia growth. All the independent experiments were conducted three times with quadruplicates at each test concentration. Ethanol solution 1% in water was the negative control and bronopol, clotrimazole, and itraconazole were the positive controls.

## Spores germination inhibition assay

The spore germination assay against *Saprolegnia* strains was performed according to the agar dilution method [23]. The minimum oomyceticidal concentration (MOC) and detailed protocols for the biological assays was defined previously [19].

## Mycelial growth inhibition assay

Inhibition of mycelial growth was assayed using the method described [23] with small modifications. Oomycete growth was measured as the colony diameter, and toxicity of the resinous exudates and the compounds **1**–**6** against *Saprolegnia* strains was measured in terms of the percentage of mycelia inhibition by a formula described in detail elsewhere [19].

## Determination of fractional inhibitory concentrations

Synergy between more bioactive compounds of resinous exudate was tested using the checkerboard microtiter assay [24, 25]. To detect a possible reduction of the MIC values of each compound when used in combination, twofold serial dilutions of one compound were tested against twofold serial dilutions of the other compound. Results were expressed as the FIC index according to the following formula.

$${\text{FIC}}\, = \,\left( {\text{A}} \right)/{\text{MIC}}_{\text{A}} \, + \,\left( {\text{B}} \right)/{\text{MIC}}_{{{\text{B}}.}}$$

where, MIC_A_ and MIC_B_ are the MICs of compounds A and B tested alone, and where (A) and (B) are the MICs of the two compounds tested in combination. An FIC index of 0.5 indicates strong synergy (representing the equivalent of a fourfold decrease in the MIC of each compound tested), while an FIC index of 1.0 indicates that the antimicrobial activity of the two compounds are additive (i.e. a twofold decrease in the MIC of each compound tested).

## Statistical analysis

Determinations of MIC, MOC, cellular leakage, MGI, and FIC were performed in triplicate and the results are expressed as mean values ± SD. The results were analyzed by the standard method [19].

## Results

**Fig. 1:**
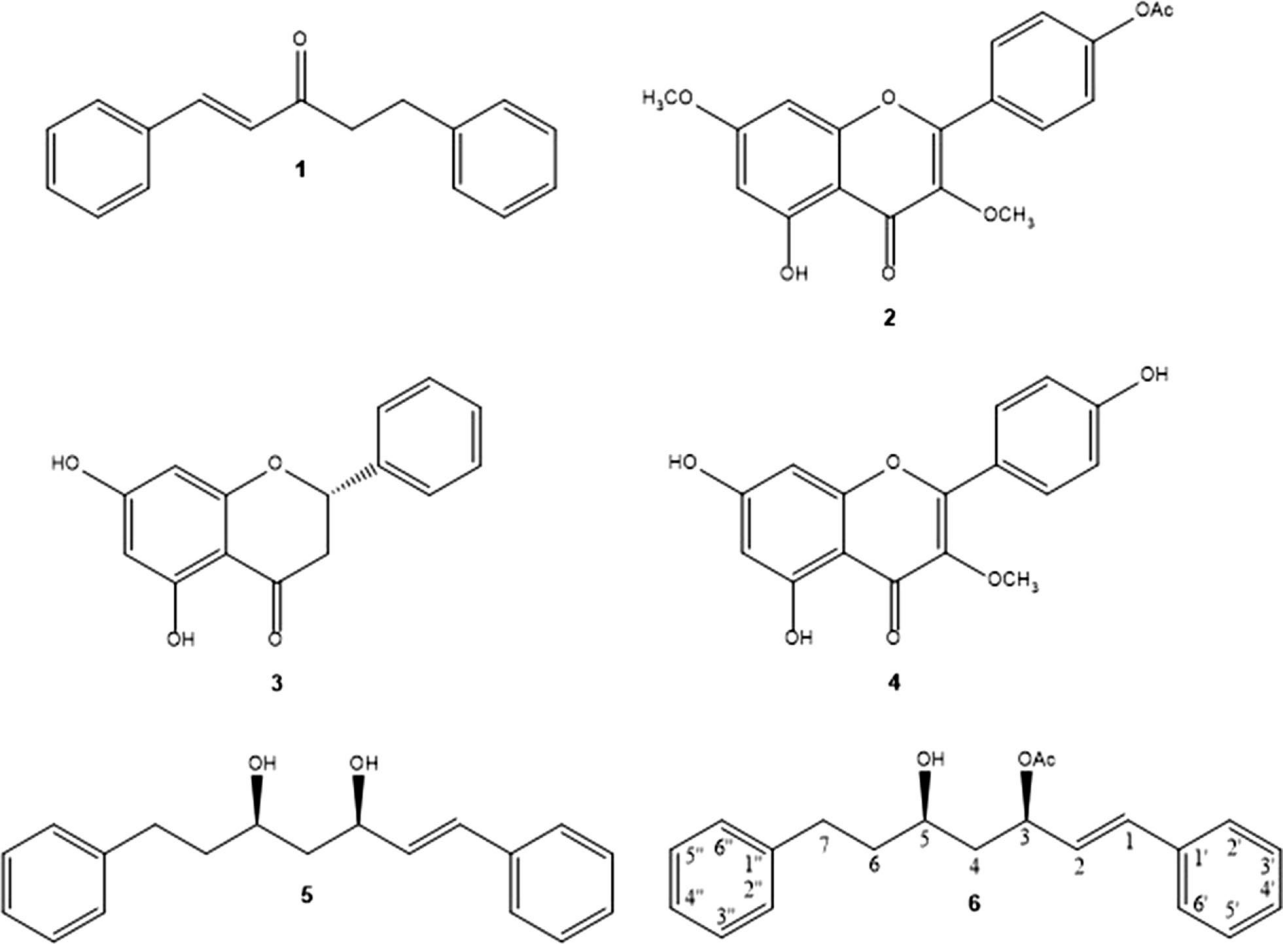
Structures of natural compounds **1**–**6** from *E. illinita*

Searching for novel bioactive substances from medicinal plant *E. illinita* against strains of *Saprolegnia parasitica* and *S. australis*, five known compounds (**1**–**5**) were isolated from the resinous exudate of *E. illinita* by using various chromatographic methods, with one new acetylated diarylheptanoid, (3*S*,5*S*)-(*E*)-5-hydroxy-1,7-diphenylhept-1-en-3-yl acetate (**6**) (Fig. 1). The structures of the known compounds 1,5-diphenylpent-1-en-3-one (**1**), 4-(5-hydroxy-3,7-dimethoxy-4-oxo-4*H*-chromen-2-yl)phenyl acetate (**2**), pinocembrin (**3**), kaempferol 3-*O*-methylether (**4**), (3*S*,5*S*)-(*E*)-1,7-diphenylhept-1-ene-3,5-diol (**5**) were determined by comparison to the ^1^H- and ^13^C-NMR spectral data in the literatures [26–30].

Compound **6** was isolated as a pale yellow solid of molecular formula C_21_H_24_O_3_. The ^1^H and ^13^C NMR spectra of **6** were very similar to those of **5**. However, the ^1^H NMR spectrum of **6** indicated the presence of two phenyl groups (*δ*: 7.36–7.17 ppm, 10 H), a pair of trans olefinic protons (*δ*: 6.55 and 6.07 ppm, *J *= 15.8 Hz), one proton of acetylated methine (*δ*: 5.68 ppm) and one hydroxylated methine (*δ*: 3.53 ppm). One of olefinic protons (*δ*: 6.07 ppm) was coupled with the acetylated methine proton (*δ*: 5.68 ppm). In addition, the ^1^H NMR spectrum showed that the hydroxylated and acetylated protons are neighbors to the protons at *δ*: 1.69–1.78 ppm, not the protons at *δ*: 2.65–2.81 ppm. The ^13^C NMR spectrum of the compound **6** indicated the presence of three methylenes (*δ*: 32.1, 38.6 and 43.3 ppm), one (*δ*: 66.6 ppm) hydroxylated methine and one (*δ*: 68.6 ppm) acetylated methine, a carbonyl group (*δ*: 171.7 ppm), a methyl group (*δ*: 21.1 ppm), two unhydrogenated sp^2^-carbons (*δ*: 129.4 and 131.6 ppm), and twelve sp^2^-carbons bearing a hydrogen. The structure of compound **6** was unequivocally assigned from 2D HSQC and HMBC spectra data. Thus, for compound **6**, the signals at *δ*_H_: 6.55 ppm (d, *J *= 15.8 Hz, 1H, H-1) showed ^*3*^*J*_H–C_ HMBC correlations with C-2′ and C-6′ (*δ*_C_: 128.6 ppm) and C-3 (*δ*_C_: 68.6 ppm) and ^*2*^*J*_H–C_ correlation with C-1′ (*δ*_C_: 135.9 ppm) and C-2 (*δ*_C_: 127.6 ppm) also were observed. Thus, the structure of **6** was concluded to be *trans*-5-hydroxy-1,7-diphenylhept-1-en-3-yl acetate. This conclusion was also supported by saponification of **6** with sodium carbonate to afford the diol derivate **5**, which gave the same spectral data. Thus, compound **6** was unambiguously assigned the depicted structure (see Additional file 1).

Anti-oomycete activity of the resinous exudate obtained from *E. illinita* against *S. parasitica* and *S. australis* in different concentrations was expressed as the minimum inhibitory concentrations (MIC), the minimum oomycidal concentrations (MOC) and the membrane damage (Table 1). Table 1 showed that the resinous exudate exhibited strong activity against both strains, with MIC and MOC values of 75 µg/mL. Here, the membrane damage percentage of resinous exudate was 72% for *S. parasitica* and 75% for *S. australis*, thus demonstrating the potency of *E. illinita* as anti-oomycete agent. Therefore, the resinous exudate could become a very important natural anti-oomycete agent. In addition, a comparison with a commercial oomycide (Bronopol) that provides total inhibition at 175 µg/mL suggests that the anti-saprolegnia activity of *E. illinita* resin is comparable (Tables 1 and 2). Thus, to reduce chemical inputs, the resinous exudate of *E. illinita* could constitute a complementary strategy to the use of pesticides against downy mildew.

**Table 1:** Minimum inhibitory concentrations (MIC), Minimum oomycidal concentrations (MOC) and damage values of compounds **1**–**6** against *S. parasitica* and *S. australis*

Compound	MIC (µg/mL)		MOC (µg/mL)		Damage (%)^a^	
Resin	75	75	75	75	72	75
**1**	100	100	125	125	50	53
**2**	> 200	200	> 200	> 200	0	0
**3**	125	125	150	150	40	43
**4**	> 200	> 200	> 200	> 200	0	0
**5**	200	200	> 200	> 200	0	0
**6**	50	50	75	75	73	76
Bronopol	175	175	> 200	175	36	30
Safrole	150	150	> 200	200	38	33
Eugenol	150	150	> 200	175	31	38
Fluconazole	> 200	200	> 200	> 200	0	0
Ketoconazole	200	200	200	200	0	0
SDS	–	–	–	–	100	100

^a^Damage produced by compounds **1**–**6** compared to the damaged produced by the sodium dodecyl sulfate (SDS). SDS was utilized at a final concentration of 2% that produces a 100% of cell lysis. The assay was performed in duplicates

**Table 2:** Mycelial growth inhibition (MGI) values of compounds **1**–**6** against *S. parasitica* and *S. australis* at 48 h

Compound	MGI (µg/mL)^a^	
	*S. parasitica*	*S. australis*
Resin	100	100
1	33	35
2	0	0
3	33	36
4	0	0
5	0	0
6	100	100
Bronopol	0	35

^a^MGI values calculated for 200 µg/mL of each compound

To explain its anti-oomycete activity, the main compounds of resinous exudate were tested against *Saprolegnia sp.* The compounds with the ability to inhibit *S. parasitica* and *S. australis* development (MIC and MOC values) were compound **6** (50 and 75 µg/mL respectively), compound **1** (100 and 125 µg/mL respectively), and pinocembrin **3** (125 and 150 µg/mL respectively). Furthermore, membrane damage caused by compounds **1** and **3** varied between 40 and 50% for *S. parasitica* and 43–53% for *S. australis*; in contrast, compound **6** exerted most membrane damage for both *Saprolegnia* strains (Table 1). The other compounds did not present inhibitory effects.

Then, the effects on sporulation were assessed by exposing mycelial colonies to resinous exudates and natural compounds and the number of zoospores released was calculated after 48 h (Table 2). The results of this assay confirmed effectiveness of *E. illinita* resinous exudates and compound **6**, **1** and **3** against both pathogenic strains, as compared to the other compounds and a positive control, such as bronopol, fluconazole, ketoconazole, and safrole [8, 19, 31]. These results are in agreement with those described by other authors. Indeed, the new diarylheptanoid **6** belongs to the family of linear diarylheptanoids which have been isolated from various sources, can be easily synthesized, and have shown diverse biological activities [32]. In addition, the lipophilicity of acetate unit appears to be another important factor for anti-oomycete activity of the compound **6** where the inhibition activity decreased for dihydroxylate **5**, which is inactive against *Saprolegnia* [33]. The compound **1** presents a structural analogue which has been isolated from *Stellera chamaejasme* L., and which showed good insecticidal property and antifeedant activity [34]. The flavonoid pinocembrin **3** also possesses antifungal property and anti-oomycete activity against *Penicillium italicum* and *Candida albicans* and *Plasmopara viticola* [35, 36].

Finally, the synergistic antimicrobial activity against *Saprolegnia* strains between the most active compound **6** and the other active compounds (**1** and **3**) was determined (Table 3). Interestingly, strong synergistic anti-oomycete activity was observed between the compounds **6** and **1** (FIC = 0.25), and with compound **3** an additive effect was observed (FIC = 1.0).

**Table 3:** Synergistic effect of most active compound **6** against *Saprolegnia* strains

*Saprolegnia* strain	FIC index^a^	
	**1 **+** 6**	**6 **+** 3**
*S. parasitica*	0.25	1.0
*S. australis*	0.25	1.0

^a^FIC index were interpreted as follows: ≤ 0.5, strong synergy; 0.5–1, synergy; ≥ 1, additive effect; ≥ 2, antagonism

Therefore, the significant anti-oomycete effect of resinous exudate is most evidently due to the presence of compound **6** in the exudates, which acts synergistically with the other compounds (**1** and **3**) against *S. parasitica* and *S. australis*. In brief, the results of the synergistic effects of compound **6** reflect its central role in resinous exudate effectiveness against *Saprolegnia* strains.

## Conclusions

In summary, six compounds were isolated and characterized from *E. illinita* resinous exudates, including two hemisynthetic pinocembrin compounds. Furthermore, one new molecule was isolated for the first time from the resinous exudates of *E. illinita*: (3*S*,5*S*)-(*E*)-5-hydroxy-1,7-diphenylhept-1-en-3-yl acetate (**6**). Significant antioomycete activities in *E. illinita* resin and novel natural compound **6** were observed against *S. parasitica* and *S. australis*. Based on these results, resinous exudates continue to spark scientific interest in chemistry due to the presence of bioactive metabolites; as an alternative solution to current pathologies; and, from a commercial point of view, due to fast processing and low required investment.

## Additional file

**Additional file 1.** **Figure S1.** ^1^H-NMR spectrum (400 MHz, CDCl_3_) of compound **6**. **Figure S2.** ^13^C-NMR spectrum (100 MHz, CDCl_3_) of compound **6**. **Figure S3.** DEPT 135 º NMR spectrum (100 MHz, CDCl_3_) of compound **6**. **Figure S4.** ^1^H-^13^C-HSQC NMR spectrum of compound **6**. **Figure S5.** ^1^H-^13^C-HMBC NMR spectrum of compound **6**. **Figure S6.** HRMS spectrum of compound **6**.
