# Supplementary material for: Isolation and identification of compounds from the resinous exudate of Escallonia illinita Presl. and their anti-oomycete activity
Source: BMC Chem. 2019 Jan 28;13(1):1. doi: 10.1186/s13065-019-0516-8 (PMC6659570; doi:10.1186/s13065-019-0516-8)

**Additional file 1**

Isolation and identification of compounds from the resinous exudate of *Escallonia illinita* Presl. and their anti-oomycete activity

Iván Montenegro ^1^, Elizabeth Sánchez ^2^, Enrique Werner ^3^, Patricio Godoy ^4^, Yusser Olguín ^5^, Nelson Caro ^6^, Nicole Ehrenfeld ^6^, and Alejandro Madrid ^7^,*

^1^ Escuela de Obstetricia y Puericultura, Facultad de Medicina, Universidad de Valparaíso, Angamos 655, Reñaca, Viña del Mar 2520000, Chile; **ivan.montenegro@uv.cl**

^2^ Centro de Biotecnología, Dr. Daniel AlKalay Lowitt, Universidad Técnica Federico Santa María, Avda. España 1680, Valparaíso 2340000, Chile; **elizabeth.sanchez@usm.cl**

^3^ Departamento De Ciencias Básicas, Campus Fernando May Universidad del Biobío. Avda. Andrés Bello s/n casilla 447, Chillán 3780000, Chile;

**ewerner@ubiobio.cl**

^4^ Instituto de Microbiología Clínica, Facultad de Medicina, Universidad Austral de Chile, Los Laureles s/n, Isla Teja, Valdivia 5090000, Chile;

**patricio.godoy@uach.cl**

^5^ Center for Integrative Medicine and Innovative Science (CIMIS), Facultad de Medicina, Universidad Andrés Bello, Santiago 8320000, Chile;

**yusser.olguin@unab.cl**

^6^ Centro de Investigación Australbiotech, Universidad Santo Tomás, Avda. Ejército 146, Santiago 8320000, Chile;

**ncaro@australbiotech.cl; nicole.ehrenfeld@australbiotech.cl**

^7^ Departamento de Química, Facultad de Ciencias Naturales y Exactas, Universidad de Playa Ancha, Avda. Leopoldo Carvallo 270, Playa Ancha, Valparaíso 2340000, Chile; **alejandro.madrid@upla.cl**

*** Correspondence: alejandro.madrid@upla.cl; Tel.: +56-032-250-0526**

List of Additional Information

Figure-S1: ^1^H-NMR spectrum (400 MHz, CDCl_3_) of compound **6**

Figure-S2: ^13^C-NMR spectrum (100 MHz, CDCl_3_) of compound **6**

Figure-S3: DEPT 135 º NMR spectrum (100 MHz, CDCl_3_) of compound **6**

Figure-S4: ^1^H-^13^C-HSQC NMR spectrum of compound **6**

Figure-S5: ^1^H-^13^C-HMBC NMR spectrum of compound **6**

Figure-S6: HRMS spectrum of compound **6**

Figure-S1: ^1^H-NMR spectrum (400 MHz, CDCl_3_) of compound **6**

**
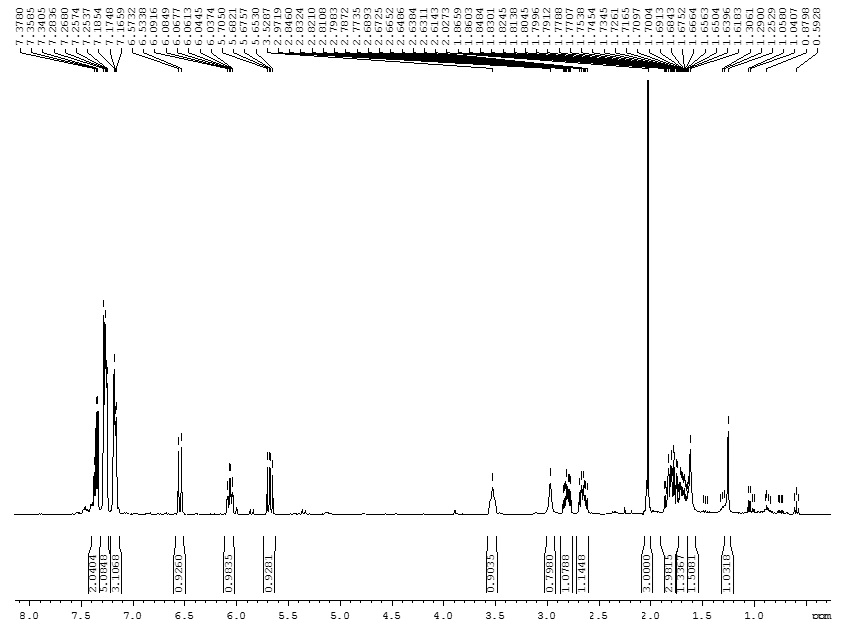
**

Figure-S2: ^13^C-NMR spectrum (100 MHz, CDCl_3_) of compound **6**

Figure-S3: DEPT 135 º NMR spectrum (100 MHz, CDCl_3_) of compound **6**

Figure-S4: ^1^H-^13^C-HSQC NMR spectrum of compound **6**


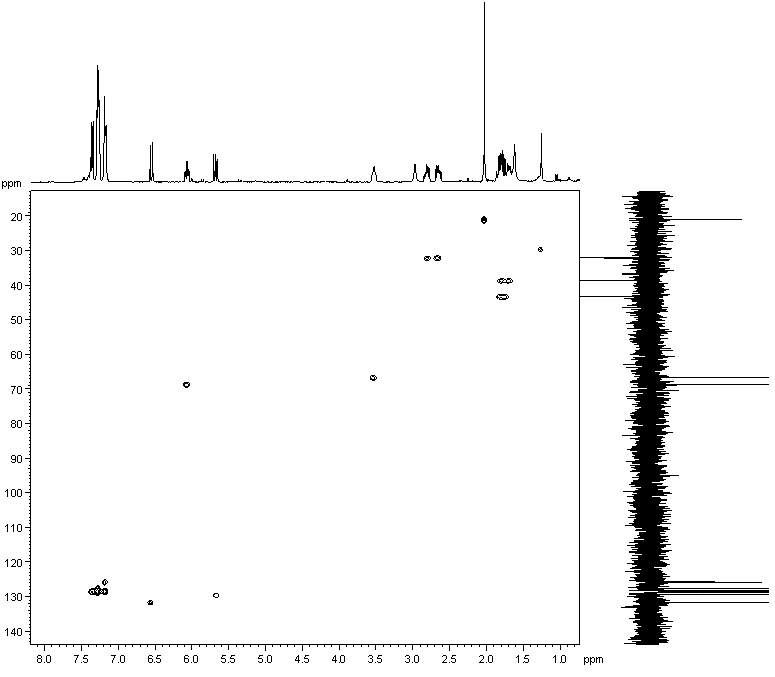


Figure-S5: ^1^H-^13^C-HMBC NMR spectrum of compound **6**


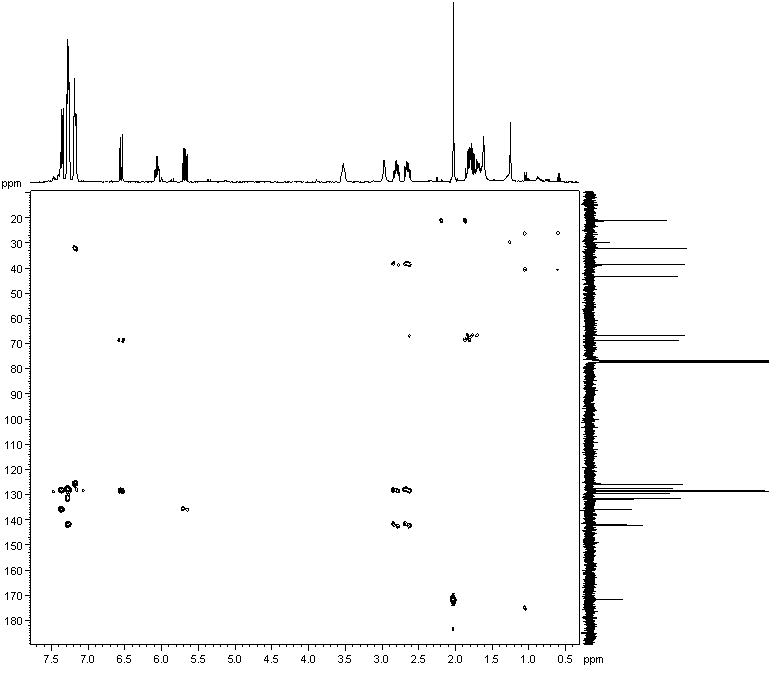


Figure-S6: HRMS spectrum of compound **6**


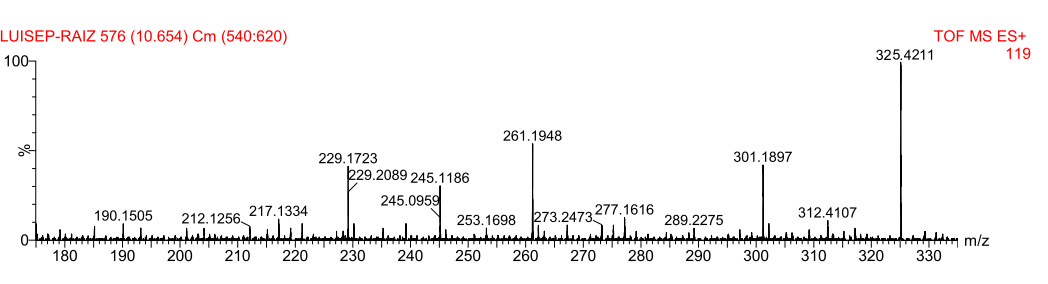

Supplement: Supplementary file 1 — Additional file 1. Figure S1. 1H-NMR spectrum (400 MHz, CDCl3) of compound 6. Figure S2. 13C-NMR spectrum (100 MHz, CDCl3) of compound 6. Figure S3. DEPT 135 º NMR spectrum (100 MHz, CDCl3) of compound 6. Figure S4. 1H-13C-HSQC NMR spectrum of compound 6. Figure S5. 1H-13C-HMBC NMR spectrum of compound 6. Figure S6. HRMS spectrum of compound 6. [file 13065_2019_516_MOESM1_ESM.doc]
